# Unfolding dermatologic spectrum of Behçet’s disease in Italy: real-life data from the International AIDA Network Behçet’s disease Registry

**DOI:** 10.1007/s11739-023-03410-9

**Published:** 2023-10-06

**Authors:** Martina D’Onghia, Elisa Cinotti, Alessandra Cartocci, Antonio Vitale, Valeria Caggiano, Linda Tognetti, Francesca La Marca, Jurgen Sota, Stefano Gentileschi, Giovanni Rubegni, Giuseppe Lopalco, Silvana Guerriero, Marcello Govoni, Sara Monti, Piero Ruscitti, Fabrizio Angeli, Francesco Carubbi, Roberto Giacomelli, Francesco Ciccia, Matteo Piga, Giacomo Emmi, Stefania Costi, Gian Domenico Sebastiani, Florenzo Iannone, Veronica Spedicato, Giovanni Alessio, Francesca Ruffilli, Alessandra Milanesi, Martina Gentile, Francesca Crisafulli, Alessia Alunno, Luca Navarini, Daniela Iacono, Alberto Cauli, Francesca Ricci, Carla Gaggiano, Maria Tarsia, Elena Bartoloni, Giovanni Conti, Ombretta Viapiana, Francesca Li Gobbi, Amato de Paulis, Paola Parronchi, Emanuela Del Giudice, Patrizia Barone, Alma Nunzia Olivieri, Emanuele Bizzi, Maria Cristina Maggio, Alberto Balistreri, Bruno Frediani, Gian Marco Tosi, Claudia Fabiani, Pietro Rubegni, Luca Cantarini

**Affiliations:** 1 Unit of Dermatology, Department of Medical, Surgical and Neurological Science, University of Siena, Siena, Italy; 2 Department of Medical Biotechnologies, University of Siena, Siena, Italy; 3 Department of Medical Sciences, Surgery and Neurosciences, Research Center of Systemic Autoinflammatory Diseases and Behçet’s Disease Clinic, University of Siena, Siena, Italy; 4 Azienda Ospedaliero-Universitaria Senese [European Reference Network (ERN) for Rare Immunodeficiency, Autoinflammatory and Autoimmune Diseases (RITA) Center] Siena, Siena, Italy; 5 Department of Ophthalmology, University of Catania, 9298 Catania, Italy; 6 Department of Precision and Regenerative Medicine and Ionian Area (DiMePRe-J), Policlinic Hospital, University of Bari, Bari, Italy; 7 Department of Ophthalmology and Otolaryngology, University of Bari, Bari, Italy; 8 Rheumatology Unit, Department of Medical Sciences, Azienda Ospedaliero-Universitaria S. Anna-Ferrara, University of Ferrara, Ferrara, Italy; 9 Rheumatology Department, IRCCS Policlinico S. Matteo Fondazione, University of Pavia, Pavia, Italy; 10 Rheumatology Unit, Department of Biotechnological and Applied Clinical Sciences, University of L’Aquila, L’Aquila, Italy; 11 Rheumatology and Clinical Immunology, Spedali Civili and Department of Clinical and Experimental Sciences, University of Brescia, Brescia, Italy; 12 Department of Life, Health & Environmental Sciences and Internal Medicine and Nephrology Unit, Department of Medicine, University of L’Aquila and ASL Avezzano-Sulmona-L’Aquila, San Salvatore Hospital, L’Aquila, Italy; 13 Clinical and Research Section of Rheumatology and Clinical Immunology, Fondazione Policlinico Campus Bio-Medico, Via Álvaro del Portillo 200, 00128 Rome, Italy; 14 Rheumatology and Clinical Immunology, Department of Medicine, School of Medicine, University of Rome “Campus Biomedico”, Rome, Italy; 15 Department of Precision Medicine, Università Degli Studi Della Campania Luigi Vanvitelli, Naples, Italy; 16 Rheumatology Unit, Department of Medical Sciences, University and AOU of Cagliari, Cagliari, Italy; 17 Department of Experimental and Clinical Medicine, University of Florence, Florence, Italy; 18 Department of Medicine, Centre for Inflammatory Diseases, Monash Medical Centre, Monash University, Melbourne, Australia; 19 Unit of Pediatric Rheumatology, ASST Gaetano Pini-CTO, Milan, Italy; 20 U.O.C. Reumatologia, Ospedale San Camillo-Forlanini, Rome, Italy; 21 Pediatric Clinic, University of Brescia and Spedali Civili Di Brescia, Brescia, Italy; 22 Rheumatology Unit, Department of Medicine, University of Perugia, Perugia, Italy; 23 Pediatric Nephrology and Rheumatology Unit, AOU Policlinic G Martino, Messina, Italy; 24 Rheumatology Unit, University of Verona, Policlinico G. B. Rossi, Verona, Italy; 25 Rheumatology Unit, San Giovanni di Dio Hospital, Florence, Italy; 26 Department of Translational Medical Sciences, Section of Clinical Immunology, University of Naples Federico II, Naples, Italy; 27 Center for Basic and Clinical Immunology Research (CISI), World Allergy Organization (WAO) Center of Excellence, University of Naples Federico II, Naples, Italy; 28 Department of Maternal Infantile and Urological Sciences, Polo Pontino, Sapienza University of Rome, Rome, Italy; 29 Department of Clinical and Experimental Medicine, University of Catania, Catania, Italy; 30 Department of Woman, Child and of General and Specialized Surgery, University of Campania “Luigi Vanvitelli”, Naples, Italy; 31 Medicina Interna, Ospedale Fatebenefratelli, Milan, Italy; 32 University Department of Health Promotion, Mother and Child Care, Internal Medicine and Medical Specialties (PROMISE) “G. D’Alessandro”, University of Palermo, Palermo, Italy; 33 Bioengineering and Biomedical Data Science Lab, Department of Medical Biotechnologies, University of Siena, Siena, Italy; 34 Ophthalmology Unit, Department of Medicine, Surgery and Neurosciences, University of Siena, Siena, Italy; 35 Research Center of Systemic Autoinflammatory Diseases and Behçet’s Disease Clinics, Department of Medical Sciences, Surgery and Neurosciences, Rheumatology Unit, University of Siena, Policlinico “Le Scotte”, Viale Bracci 16, 53100 Siena, Italy

**Keywords:** Aphthosis, Pseudofolliculitis, Erythema nodosum, Diagnosis, Autoinflammatory diseases

## Abstract

Behçet’s disease (BD) is a heterogeneous multifactorial autoinflammatory disease characterized by a plethora of clinical manifestations. Cutaneous lesions are considered hallmarks of the disease. However, their evolution over time and a thorough description are scarcely reported in non-endemic regions. The aim of this study was to detail BD skin manifestations and their evolution over time in Italy, as well as the dermatological prognostic impact of specific cutaneous features in long-standing disease. Data were collected in a double fashion, both retrospectively and prospectively, from the AutoInflammatory Disease Alliance (AIDA) international registry dedicated to BD, between January 2022 and December 2022. A total of 458 Italian patients were included. When assessing skin manifestations course, the constant or sporadic presence or absence of cutaneous involvement between onset and follow-up was considered. Oral ulcers (OU) (88.4%) and genital ulcers (GU) (52.6%), followed by skin involvement (53.7%) represented the most common presenting mucocutaneous manifestations at disease onset. Up to the time of enrolment into the AIDA registry, 411 (93.8%) patients had suffered from OU and 252 (57.9%) from GU; pseudofolliculitis (PF) accounted for the most common skin manifestation (170 patients, 37.1%), followed by erythema nodosum (EN) (102 patients, 22.3%), skin ulcers (9 patients, 2%) and pyoderma gangrenosum (4 patients, 0.9%). A prospective follow-up visit was reported in 261/458 patients; 24/148 (16.2%) subjects with skin involvement as early as BD onset maintained cutaneous lesions for the entire period of observation, while 120 (44.1%) patients suffered from sporadic skin involvement. Conversely, 94/113 (83.2%) with no skin involvement at disease onset did not develop skin lesions thereafter. At follow-up visits, cutaneous involvement was observed in 52 (20%) patients, with a statistically significant association between PF and constant skin involvement (*p* = 0.031). BD in Italy is characterized by a wide spectrum of clinical presentations and skin manifestations in line with what is described in endemic countries. Patients with skin disease at the onset are likely to present persistent cutaneous involvement thereafter; mucocutaneous lesions observed at the onset, especially PF, could represent a warning sign for future persistent skin involvement requiring closer dermatological care.

**Supplementary Information:**

The online version contains supplementary material available at 10.1007/s11739-023-03410-9.

## Introduction

Behcet’s disease (BD) is a relapsing–remitting multifactorial inflammatory disorder of unknown origin [1]. It is characterized by mucocutaneous lesions and articular features associated with a variable degree of major organ involvement, such as ocular, gastrointestinal, neurological, and vascular manifestations [2]. The onset of BD usually occurs in the third and fourth decades of life, with a negative impact on patients’ quality of life.

Epidemiological studies highlighted an equal sex predilection, although a male preponderance in the Middle East and Mediterranean population has been described [3]. BD has a worldwide occurrence and a distinct geographic variation, with a higher distribution among the so-called "silk road", extending from Japan to the Middle East and Mediterranean Basin. The higher prevalence was reported in Turkey (420/1000000) [4], while the disease is rarely seen in Western Countries where the estimated prevalence is 0.27–7.5/100000 in Europe and 0.33–5.2/100000 in the United States [5, 6]. Although likely multifactorial, the reasons behind the disease expression in countries remain largely unknown.

Mucocutaneous lesions, especially oral ulcers (OU) and genital ulcers (GU), pseudofolliculitis (PF), and erythema nodosum (EN), are considered hallmarks of BD [7]. Skin lesions are the most common manifestations at disease onset, usually preceding other systemic involvements [8]. Hence, their recognition may allow earlier diagnosis and treatment, and better prognosis. Furthermore, skin manifestations have been found to explain the clinical heterogeneity of BD [9].

A reliable literature on skin manifestations in non-endemic regions is still lacking. Therefore, the purpose of this study was to describe BD dermatological manifestations in Italy, as a paradigm of Western countries.

## Methods

### Study design and participants

This study was based on data collected in the International AutoInflammatory Disease Alliance (AIDA) Registry dedicated to BD [10]. The enrolment of BD patients on the AIDA Registry started on January 31st, 2021, and we extrapolated information on 458 Italian BD patients enrolled in the AIDA Registry up to December 2022. BD was diagnosed according to either the International Criteria for Behçet’s Disease, or the International Study Group Criteria, or the classification criteria for pediatric BD, depending on the age at disease onset [11].

Age, sex, age at diagnosis, manifestations at disease onset, and clinical mucocutaneous features arising over time were recorded in all patients at the enrolment. Data on current medications were also recorded at the last follow-up visit included in the Registry. The course of skin manifestations in BD patients was classified according to the persistence or sporadic presence or absence of skin involvement during the whole period ranging from disease onset to the last follow-up visit. Sporadic presence of cutaneous manifestations was meant as temporary appearance of skin manifestations (i.e., observed at the onset, but no longer present over the follow-up or vice-versa).

The primary aims of the study were to describe skin involvement in BD patients from Italy, as an example of a European non-endemic country, and to report the evolution of skin manifestations over time.

The endpoints of our study consisted of i) describing the specific types of skin manifestations and the most frequently involved sites; ii) analyzing changes in the type and frequency of skin manifestations during follow-up.

### Protocol approval

The study was approved by the Ethics Committee of the University Hospital of Siena, Siena, Italy (Protocol Number 14951) as part of the AIDA Program. The study protocol conformed to the tenets of the Helsinki Declaration. Written informed consent to participate in the international AIDA Registry for BD patients was obtained from all patients and/or their legal guardians.

### Statistical analysis

Descriptive statistics included mean and standard deviation (SD), and median for continuous variables, while frequency and percent were reported for categorical variables. Chi-squared test, Fisher’s exact test and Student’s t test were performed to compare groups. A *p* < 0.05 was considered statistically significant. All data were assessed using the software R version 4.1.0.

## Results

**Fig. 1:**
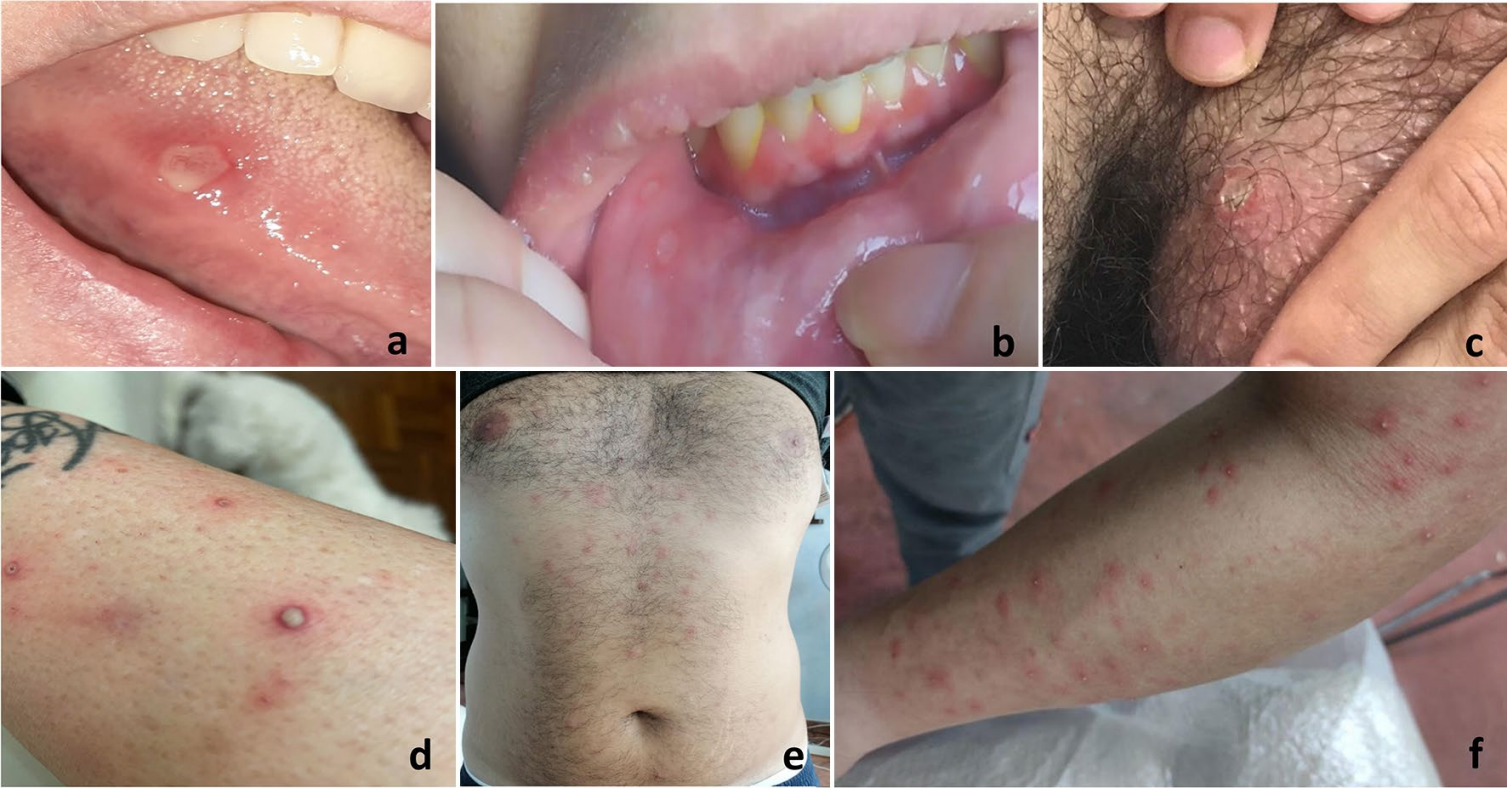
Mucocutaneous manifestations in Behcet’s disease: minor oral ulcer with a yellow base under the tongue, surrounded by an erythematous halo (**a**); multiple minor oral ulcers located on the buccal mucosa (**b**); single oval ulcer of the scrotum with erythematous borders and yellowish pseudomembranes overlying a necrotic base (**c**); typical pseudofolliculitic lesions with pustules surrounded by erythema (**d**); pseudofolliculitis localized on the trunk (**e**); pseudofolliculitis of the arm (**f**)

This study included 458 patients; the female sex corresponded to 272 (59%) subjects. The mean (± SD) age at diagnosis and at enrolment into the AIDA registry were 35.7 ± 13.6 and 44.4 ± 15.1 years, respectively. Demographic data and manifestations referring to the BD onset are summarized in Table 1, while the frequency of mucocutaneous involvement at the enrolment is listed in Table 2. As expected, OU accounted for the most common initial sign (88.4%), followed by GU (52.6%). Skin involvement since the beginning of BD onset was observed in 246 (53.7%) patients. At BD onset, arthralgia (54.4%), ocular involvement (41.3%), arthritis (36%), neurological manifestations (25.8%), unexplained fever episodes (24.9%), and gastrointestinal involvement (21.4%) represented the most common extracutaneous manifestations. At the enrolment into the AIDA Registry, 411 (93.8%) patients suffered from OU (Fig. 1a, b), 110 (48.2%) of which complained of 3–5 concomitant OU for each attack, 78 (34.2%) of 1–2 OU for each attack and 40 (17.5%) of more than 5 OU for each attack. Minors (< 10 mm in diameter), major (> 10 mm in diameter) and herpetiform OU were observed in 34.7%, 18.6%, and 2.2% of patients, respectively.

**Table 1:** Frequency of manifestations at onset

	Overall (*n* = 458) Age
Age at diagnosis in years, mean (SD)	35.74 (13.6)
Age at enrolment in years, mean (SD)	44.41 (15.1)
Disease duration at the enrolment in years, median (interquartile range)	12.7 (18.7)
Disease duration at the last visit in years, median (interquartile range)	13.7 (18.7)
Females, *n* (%)	272 (59%)
Recurrent ulceration, *n* (%)	
Oral ulcers, *n* (%)	405 (88.4)
Genital ulcers, *n* (%)	241 (52.6)
Skin involvement, *n* (%)	246(53.7)
Arthralgia, *n* (%)	249 (54.4)
Ocular involvement, *n* (%)	189 (41.3)
Articular involvement, *n* (%)	165 (36.0)
Fever of unexplained origin, *n* (%)	114 (24.9)
Gastrointestinal involvement, *n* (%)	98 (21.4)
Central neurological involvement, *n* (%)	71 (15.5)
Vascular involvement, *n* (%)	71 (15.5)
Peripheral neurological involvement, *n* (%)	47 (10.3)
Cardiac involvement, *n* (%)	11(2.4)
Psychiatric involvement, *n* (%)	8 (1.7)
Inflammatory low back pain, *n* (%)	5 (1.1)

**Table 2:** Frequency of BD mucocutaneous lesions at the time of the enrolment into the AIDA registry

	(*n* = 458)
Patients with oral ulcers, *n* (%)	411 (93.8)
Mean number of concurrent OU, *n* (%)	
1–2 ulcers	78 (34.2)
3–5 ulcers	110 (48.2)
More than 5 ulcers	40 (17.5)
Size of OU, *n* (%)	
Patients with minor aphthous ulcerations	159 (34.7)
Patients with major aphthous ulcerations	85 (18.6)
Patients with herpetiform ulcerations	10 (2.2)
Patients with genital ulcers, *n* (%)	**252 (57.9)**
Mean number of concurrent GU, *n* (%)	
1–2 ulcers	98 (70.5)
3–5 ulcers	28 (20.1)
More than 5 ulcers	13 (9.7)
Localization, *n* (%)	
Scrotum	31 (6.8)
Shaft	7 (1.5)
Glans	22 (4.8)
Perianal region	9 (2.0)
Perineum	8 (1.7)
Labia minora	33 (7.2)
Labia majora	56 (12.2)
Vagina	33 (7.2)
Skin Involvement, *n* (%)	
Patients with pseudo folliculitis	170 (37.1)
Patients with erythema nodosum	102 (22.3)
Patients with skin ulcers	9 (2.0)
Patients with pyoderma gangrenosum	4 (0.9)

*OU* oral ulcers; *GU* genital ulcers

At the enrolment, GU were found in 252 (57.9%) patients, with the majority of patients (70.5%) presenting between 1 and 2 coexisting genital lesions, while 28 (20.1%) patients showed 3 to 5 GU and 28 (20.1%) patients presented more than 5 GU. The most frequent localisation of GU was represented by labia majora in females (56/150, 37.3%), and scrotum (28/102 27.5%) in males (Fig. 1 c).

The most common skin manifestation at the time of the enrolment was PF (170/458 patients, 37.1%) (Fig. 1 d, e, f), followed by EN (102/458 patients, 22.3%), skin ulcers (9/458 patients, 2%) and pyoderma gangrenosum (4/458 patients, 0.9%).

Roughly 57% of the cohort (261/458 patients) presented at least one prospective follow-up visit. In this subgroup of patients, 148 (56.7%) subjects had suffered from skin involvement as soon as the BD onset; 24/148 (16.2%) patients maintained cutaneous lesions during the entire period of observation in a continuous fashion, 87% of these suffering from PF and 25% from EN. Conversely, 120 (44.1%) patients suffered from recurrent skin involvement in a sporadic fashion (65% with relapsing PF and 46.7% with relapsing EN). Four (1.5%) patients did not experience further mucocutaneous affections. Among the 113 patients with no skin involvement at disease onset, 94 (83.2%) never developed skin lesions during the whole follow-up, while 18 (15.9%) cases underwent a sporadic skin involvement and 1 (0.9%) patient underwent a continuous skin disease course.

During the prospective phase, characterized by a median duration of 371 days, cutaneous involvement was observed in 52 (20%) patients, with PF involving 37 (71.2%) cases and EN 8 (15.4%) cases. A statistically significant association between PF and constant skin involvement was observed (*p* = 0.031; see Table 3). *Supplementary table 1* details the distribution of mucocutaneous manifestations and concomitant treatment in 261 BD patients with at least one follow-up visit. The presence of skin manifestations at prospective follow-up visits was associated with the use of corticosteroids (*p* = 0.019), while no significant association was found between PF and corticosteroids (*supplementary table 2*).

**Table 3:** Distribution of mucocutaneous manifestations and concomitant treatments in BD patients according to any occurrence of skin involvement at (the last) follow up visit

	Overall (*n* = 261)		
	No-skin manifestation	Skin Manifestations	*p*-value
	209	52	
Pseudofolliculitis, *n* (%)	–	37 (71.2)	–
Erythema nodosum, *n* (%)	–	8 (15.4)	–
Skin ulcers, *n* (%)	–	1 (1.9)	–
Colchicine, *n* (%)	94 (45.0)	31 (59.6)	0.083
Corticosteroids, *n* (%)	73 (34.9)	28 (53.8)	0.019
Azathioprine, *n* (%)	40 (19.1)	10 (19.2)	1.000
NSAIDs, *n* (%)	11 (5.3)	5 (9.6)	0.397
Methotrexate, *n* (%)	11 (5.3)	7 (13.5)	0.075
Sulfasalazine, *n* (%)	11 (5.3)	5 (9.6)	0.397
Biologic treatments, *n* (%)	99 (49.5)	28 (57.1)	0.424
Small molecules, *n* (%)	4 (2.0)	2 (4.2)	0.707
Mean follow-up, days (mean (SD))	362.35 (157.56)	375.89 (219.87)	0.729

*NSAIDs* Non-steroidal anti-inflammatory drugs, *SD* standard deviation

## Discussion

As a multi-systemic vasculitis, BD can affect many organs, including joints, eyes, vascular, nervous, and gastrointestinal systems; nevertheless, the clinical picture that drives BD diagnosis is mainly dominated by mucocutaneous lesions [1].

Most of the information on clinical characteristics of skin involvement is related to BD endemic regions; hence, the goal of our study was to investigate BD dermatological features in Italy.

The mean age at diagnosis in Italian BD patients was 35.7 years, confirming that this disease is mostly observed between the third and the fourth decade of life and that its onset is rarer after the age of 40 [12].

In this study, we found that most BD patients suffered from OU at onset. This finding is not surprising as extensive literature demonstrated that OU are the most frequent signs of BD, observed in up to 90% of patients at diagnosis [13]. At the time of the enrolment, we observed nearly half of BD patients suffering from mucocutaneous attacks with 3–5 OU, while one-third of individuals had 1–2 OU. Most of the patients showed minor OU, while major and herpetiform types were seen with lower frequency (18.6% and even 2.2%, respectively). In regard to GU, these were prevalent in about half of the BD population, with most patients showing 1–2 contemporary genital lesions, especially located on labia majora in females, or scrotum in males. While the frequency of occurrence and clinical features of OU and GU are comparable to other reports from different countries [14], to our knowledge, this is the first wide Italian study presenting detailed characteristics of this type of lesions.

As a whole, 53.7% of the total cohort presented cutaneous manifestations at onset. This result is consistent with the average skin involvement reported from earlier BD investigations, whose frequency ranged from a minimum of 39.4% in Egypt to a maximum of 87.1% in Korea [3]. A further result emerging from the present study highlighted that PF, followed by EN, accounted for the most common skin manifestation affecting BD patients up to the time of enrolment. Those findings are consistent with extensive medical literature and with earlier Italian reports [15–17]. Moreover, we found four cases of pyoderma gangrenosum (0.9%), less frequently observed in BD.

Considering extracutaneous symptoms at onset, arthralgia, and intraocular inflammation represented the commonest findings, followed by articular inflammation and neurological involvement. Curiously, unexplained recurrent fever episodes were observed in roughly one-quarter of patients, which is an unusual finding for BD subjects. Conversely, vascular involvement was identified in 15.5% of BD patients, resembling similar results reported in other Western countries such as Germany, the United Kingdom, the United States, and Sweden [5, 6, 18, 19]. Also, we were able to confirm many clinical manifestations previously described in other Italian cohorts [15–17] and, in general, our data consistently confirm that skin involvement is a very common BD manifestation at disease onset.

The female predilection observed in our analysis is consistent with previous reports from Western Countries [5]. However, sex distribution is in contrast with those reported from Eastern and Middle Eastern countries, where a male predominance was observed [20]. In this regard, the male-to-female ratio formerly reported in Turkey has proved to decrease during the last years and an equal rate has also been reported [21]. The exact reasons underlying gender differences in BD are still largely unknown. One possible explanation might be the cultural reluctance that prevents women from seeking medical attention because of GU in Eastern countries, leading to an underestimation of the number of BD diagnoses in women, as supported by the lower prevalence of GU in these populations [3].

We tried to assess the skin manifestations course between disease onset and the last follow-up. Intriguingly, the majority of BD patients with no skin involvement at the onset did not develop skin lesions thereafter, while patients with cutaneous manifestations at onset, showed a recurrent or constant skin involvement during BD history.

Although this was not the aim of the present study, these observations might support the existence of a subset of BD patients who are more prone to develop skin manifestations [12]. In this context, mucocutaneous lesions observed at the onset could be regarded as a warning sign and a predictive factor for future skin involvement requiring a closer dermatological follow-up.

Interestingly, PF accounted for the most frequent skin manifestation reported at the disease onset in both BD patients bound to suffer from a continuous skin disease course and those developing a recurrent cutaneous course. This finding does not contradict other Italian studies and confirms previous observations that assumed pustulosis as the most frequent cutaneous lesion in BD patients from Iran, Morocco, China, and Germany [17, 22].

Given the complexity of BD, the therapeutic approach varies according to the specific organ involvement and the severity or duration of the disease. Glucocorticoids and colchicine are systemic treatments frequently prescribed to manage BD, and particularly they can be used as first-line treatment for mucocutaneous lesions [23]. Based on our results, about one-fifth of patients had skin involvement at the prospective follow-up visits. Also in this phase, PF accounted for the most frequent skin manifestation. These patients were predominantly treated with colchicine and corticosteroids, while roughly half of patients received biologic treatments (*supplementary table 2*). A positive association between skin manifestations and corticosteroids use was observed; however, no statistically significant differences were observed when comparing patients with PF according to the use of corticosteroids or colchicine therapies. This ensures the lack of a role of these treatments, especially corticosteroids, in facilitating skin pustulosis. Our data seem to confirm that cutaneous lesions tend to decrease spontaneously with the passage of time, rather than as a result of treatment strategies [24]. However, our results could also suggest that cutaneous affections during follow-up may be independent of BD current treatments and that drug use is rather aimed at controlling other types of BD manifestations, especially major organ involvement. Nonetheless, it is also plausible that a subgroup of BD patients may show skin manifestations that are not fully controlled by treatments generally employed for this type of BD affection.

The main strength of this work relies on a relatively large sample of BD Italian patients. In addition, we have provided detailed real-life evidence about mucocutaneous manifestations in a non-endemic country. However, several limitations need to be recognized. First, the retrospective component of the study is responsible for some missing data, alongside its inherited shortcomings. Secondly, we were unaware of the specific indications for treatment strategies used to manage BD Italian patients, so we could not retrieve exactly the relationship between systemic therapies and clinical manifestations, including the mucocutaneous ones. Finally, a direct comparison between Italian studies was complicated by the heterogeneity of methods employed and the lack of a consistent body of literature on BD in Italy.

## Conclusion

Our study may highlight the frequency and a thorough clinical spectrum of mucocutaneous manifestations of BD in Italy, as a paradigm of non-endemic Western countries. The early recognition and correct classification of BD-associated skin manifestations may help in predicting the course of skin affections over time and potentially detect a specific subset of patients requiring a tighter dermatological follow-up. The prospect of a better characterization of mucocutaneous involvement associated with BD may allow the development of novel prevention and treatment approaches. To this end, the dermatologist is a crucial professional figure in the context of a multidisciplinary approach that ultimately leads to a more optimal management of the disease. Future studies are needed to assess a more detailed description of the response to the different treatment strategies.

### Supplementary Information

Below is the link to the electronic supplementary material.

Supplementary file1 (DOCX 15 KB)

## Data Availability

The datasets generated during and/or analyzed during the current study are available from the corresponding author on reasonable request.
