# Supplementary material for: Unfolding dermatologic spectrum of Behçet’s disease in Italy: real-life data from the International AIDA Network Behçet’s disease Registry
Source: Intern Emerg Med. 2023 Oct 6;18(8):2245–52. doi: 10.1007/s11739-023-03410-9 (PMC10635974; doi:10.1007/s11739-023-03410-9)
Supplement: Supplementary file 1 — Supplementary file1 (DOCX 15 KB) [file 11739_2023_3410_MOESM1_ESM.docx]

***Supplementary table 1*. Frequency of skin manifestations in patients with no-skin involvement at the BD onset and patients suffering from skin affections as early as BD development. Subjects have been distinguished according to the dermatological disease course (sporadic flares versus constant skin manifestations).**

| **Overall**  **(n=261)** | | | | |
| --- | --- | --- | --- | --- |
| **No-Skin manifestation at onset, (n=113)** | | | | |
|  | No, n (%)  94 (83) | Sporadic, n (%)  18 (15) | Constant, n(%)  1(0.8) | *p*-value |
| Pseudofollicolitis | - | 13 (72.2) | 1 (100.0) | 1.00 |
| Erythema nodosum | - | 2 (11.1) | 1 (100.0) | 0.158 |
| **Skin manifestation at onset, (n=148)** | | | | |
|  | No, n (%)  4(3) | Sporadic, n(%)  120 (81) | Constant  24(16) | p |
| Pseudofollicolitis | - | 78 (65.0) | 21 (87.5) | 0.031 |
| Erythema nodosum | - | 56 (46.7) | 6 (25.0) | 0.070 |

***Supplementary table 2*. Frequency of treatment strategies used in patients suffering from pseudo-folliculitis (PF) and those without PF during the prospective phase. Abbreviations: NSAIDs**, Non-steroidal anti-inflammatory drugs; **PF**, pseudofolliculitis.

|  | **No-PF**  **(n=15)** | **PF**  **(n=37)** | ***p*-value** |
| --- | --- | --- | --- |
| Systemic NSAIDs on demand, n (%) | 1 (6.7) | 4 (10.8) | 1.000 |
| Corticosteroids, n (%) | 8 (53.3) | 20 (54.1) | 1.000 |
| Colchicine, n (%) | 8 (53.3) | 23 (62.2) | 0.783 |
| Azathioprine, n (%) | 3 (20.0) | 7 (18.9) | 1.000 |
| Sulfasalazine, n (%) | 3 (20.0) | 2 (5.4) | 0.272 |
| Methotrexate, n (%) | 2 (13.3) | 5 (13.5) | 1.000 |
| Biologic treatments, n (%) | 9 (64.3) | 19 (54.3) | 0.749 |
| Small molecules, n (%) | 1 (6.7) | 1 (3.0) | 1.000 |
